# Comparison between RFLP and MIRU-VNTR Genotyping of *Mycobacterium tuberculosis* Strains Isolated in Stockholm 2009 to 2011

**DOI:** 10.1371/journal.pone.0095159

**Published:** 2014-04-14

**Authors:** Jerker Jonsson, Sven Hoffner, Ingela Berggren, Judith Bruchfeld, Solomon Ghebremichael, Alexandra Pennhag, Ramona Groenheit

**Affiliations:** 1 The Public Health Agency of Sweden (former Swedish Institute for Communicable Disease Control), Solna, Sweden; 2 Infectious Diseases Unit, Karolinska University Hospital, Stockholm, Sweden; 3 Department of Communicable Disease Control and Prevention, Stockholm County Council, Stockholm, Sweden; Institut Pasteur de Lille, France

## Abstract

Our aim was to analyze the difference between methods for genotyping of *Mycobacterium tuberculosis* complex isolates. We collected genotyping results from Restriction Fragment Length Polymorphism (RFLP) and Mycobacterial Interspersed Repetitive Units - Variable Numbers of Tandem Repeat (MIRU-VNTR) in a geographically limited area (Stockholm) during a period of three years. The number and proportion of isolates belonging to clusters was reduced by 45 and 35% respectively when combining the two methods compared with using RFLP or MIRU-VNTR only. The mean size of the clusters was smaller when combining methods and smaller with RFLP compared to MIRU-VNTR. In clusters with confirmed epidemiological links RFLP coincided slightly better than MIRU-VNTR but where there was a difference, the variation in MIRU-VNTR pattern was only in a single locus. In isolates with few IS*6110* bands in RFLP, MIRU-VNTR differentiated the isolates more, dividing the RFLP clusters. Since MIRU-VNTR is faster and less labour-intensive it is the method of choice for routine genotyping. In most cases it will be sufficient for epidemiological purposes but true clustering might still be considered if there are epidemiological links and the MIRU-VNTR results differ in only one of its 24 loci.

## Introduction

Tuberculosis (TB) in Sweden is mainly restricted to risk groups, in particular immigrants from high incidence countries. From 2009 to 2011, a total of 1872 cases were reported in all of Sweden and 86% were born in another country. Of the 242 cases younger than 19 years, 28 were born in Sweden and 26 of these had at least one parent born in a high incidence country. The majority of TB cases diagnosed in Sweden are infected outside of Sweden. Since most of them are in an economically productive age they have many social contacts and this makes early treatment and thorough contact tracing very important to avoid transmission of TB.

In order to monitor signs of domestic spread, genotyping by Restriction Fragment Length Polymorphism (RFLP) to investigate *Mycobacterium tuberculosis (Mtb)* complex isolates belonging to clusters has been performed at the Swedish Institute for Communicable Disease Control (SMI) since 1994. The national coverage of genotyping is currently around 85% of all cases with culture verified TB. In the county of Stockholm the coverage of genotyping is almost 100% of all culture positive cases. From 2009 to 2011, 75% of the reported cases in Stockholm were verified by a positive culture. In 2012, the reference genotyping technique was changed to Mycobacterial Interspersed Repetitive Units - Variable Numbers of Tandem Repeat (MIRU-VNTR) with additional genotyping performed on *Mtb* strains of the Beijing lineage. Since the methods are different, isolates belonging to clusters according to MIRU-VNTR will not always cluster when analyzed with RFLP and vice versa. An ideal method would produce genotyping results within the first few months of treatment while contact tracing is ongoing, to help clarify where the patient was most likely infected. This could then in a timely manner aid in the identification of others who are at risk of developing active TB.

Insertion sequence (IS) *6110* RFLP, the previous gold standard for genotyping *Mtb* complex isolates, has been extensively used for TB epidemiological studies because of its high discriminatory power (DP). However, the method is labor-intensive, time-consuming and is limited by the need for a large quantity of pure genomic DNA and its inability to discriminate between strains with low IS copy numbers. This has led to the development of a PCR-based typing method where 24 loci containing variable numbers of tandem repeat DNA elements called mycobacterial interspersed repetitive units (MIRU-VNTR) are analyzed [1]. The turn-around-time compared to RFLP is greatly reduced and the results are provided in a format easily exchangeable between different TB surveillance centers. Another PCR-based method frequently used due to its technical simplicity is spoligotyping which has a relatively low discriminatory power when used on its own [2], [3]. Twenty-four loci MIRU-VNTR combined with spoligotyping, has shown to be comparable to RFLP in detecting clusters of transmission in European population-based studies [4].

Within the frame of the EU TB PAN-NET project, a European network for the study and clinical management of TB drug resistance, *Mtb* complex isolates from cases in Stockholm have been analyzed with both MIRU-VNTR and RFLP in combination with spoligotyping between the years 2009–2011. In this study we compared the results between the different methods and then correlated this to the epidemiological data available on the cases in Stockholm to obtain a better understanding of the variability of clustering with different methods.

## Materials and Methods

### Ethics Statement

The Swedish County medical offices send clinical *Mtb* complex isolates to SMI for disease surveillance. Epidemiological data is also collected but is not always complete. In order to access medical files to collect more complete epidemiological data when needed, ethical approval was applied for. Since the study was retrospective, these were all historical cases already cured and we assumed that accessing their medical files to complete epidemiological data would not affect them in any way. The data was de-identified before analysis. Ethical approval for the study was granted. (Regional ethics committee Stockholm, number 2012/2012–13). The Regional ethics committee Stockholm waived the need to obtain written informed consent from the patients to conduct the study.

### Demographic and Clinical Information

Reporting of TB in Sweden is mandatory according to the Communicable disease act. Demographic and clinical data of culture-verified TB cases was collected from the web-based reporting system, SmiNet, and from medical records.

### Bacterial Isolates

All *Mtb* complex isolates obtained from patients with pulmonary or extra-pulmonary TB residing in Stockholm County during the years 2009–2011 were studied. These isolates were part of the TB PAN-NET project as previously described.

### Drug Susceptibility Testing

The isolates were tested for susceptibility to the first-line drugs isoniazid (INH), rifampicin (RIF), ethambutol (EMB) and pyrazinamide (PZA) using the MGIT 960 liquid culture and drug susceptibility testing systems according to the instructions of the manufacturer. The TB laboratory at the Karolinska University hospital in Stockholm performing the drug susceptibility testing, takes part in the external quality assurance program for drug susceptibility testing of *Mtb* complex isolates offered by the Swedish TB reference laboratory at SMI.

### Molecular Epidemiological Typing Techniques

The isolates were cultured on Löwenstein Jensen medium and spoligotyping was used to characterize the polymorphic direct repeat region of the *Mtb* chromosome [5]. RFLP typing was performed using the insertion sequence IS*6110* as a probe and *Pvu*II as the restriction enzyme [6], [7]. Finally, standardized 24-loci MIRU-VNTR [1] was performed using the MIRU-VNTR typing kit (Genoscreen, Lille, France). The PCR products were run with 1200 LIZ size standard on ABI3131×l sequencers with 16 capillaries. Sizing of the PCR-fragments and assignments of MIRU-VNTR alleles were done with the GeneMapper software version 4.1 (Applied Biosystems) according to the instructions of the manufacturer. External quality assurance for MIRU-VNTR was provided by the European Centre for Disease Prevention and Control (ECDC) together with the Dutch National Institute for Public Health and the Environment (RIVM) as well as by the TB PAN-NET project.

### Computer Analysis

The spoligotyping patterns, IS*6110* RFLP fingerprints and MIRU-VNTR typing results were analyzed by the BioNumerics software version 6.6 (Applied Maths, Kortrijk, Belgium). The IS*6110* RFLP fingerprints were digitalized and compared by the unweighted pair group method of arithmetic averaging (UPGMA) using the Jaccard coefficient. MIRU-VNTR data were imported from Excel spreadsheets and analyzed in BioNumerics using the categorical coefficient and similarity trees were calculated using the UPGMA. Clusters were defined as strains showing either identical IS*6110* RFLP (the same number of IS*6110* bands at identical positions) and spoligotyping patterns or identical MIRU-VNTR and spoligotyping patterns. The spoligotypes were also analyzed using the international MIRU-VNTR*plus* database website (http://www.miru-vntrplus.org/), allocated to Spoligotype International Types (SIT) and assigned to major phylogenetic lineages. In order to compare the difference in discriminatory power between the genotyping methods and the combinations of them in our material, we calculated the Hunter-Gaston index (HGI) of discriminatory ability [8].

### Epidemiological Analysis

Epidemiological data and information on contact tracing was available for all cases. A cluster was defined as two or more *Mtb* complex strains showing either identical spoligotyping patterns and RFLP or identical spoligotyping patterns and MIRU-VNTR, but will hereafter be referred to as only RFLP or MIRU-VNTR clusters. The isolates forming clusters with RFLP versus MIRU-VNTR were compared with data on drug-susceptibility, the available epidemiological data and results from contact tracing, in order to evaluate which method could best confirm epidemiological links. Epidemiological data were classified as follows:

- true link; family or social contact (friends/colleagues)
- possible link; the same geographical origin
- no link; neither of the above and no other known link.

## Results

**Figure 1:**
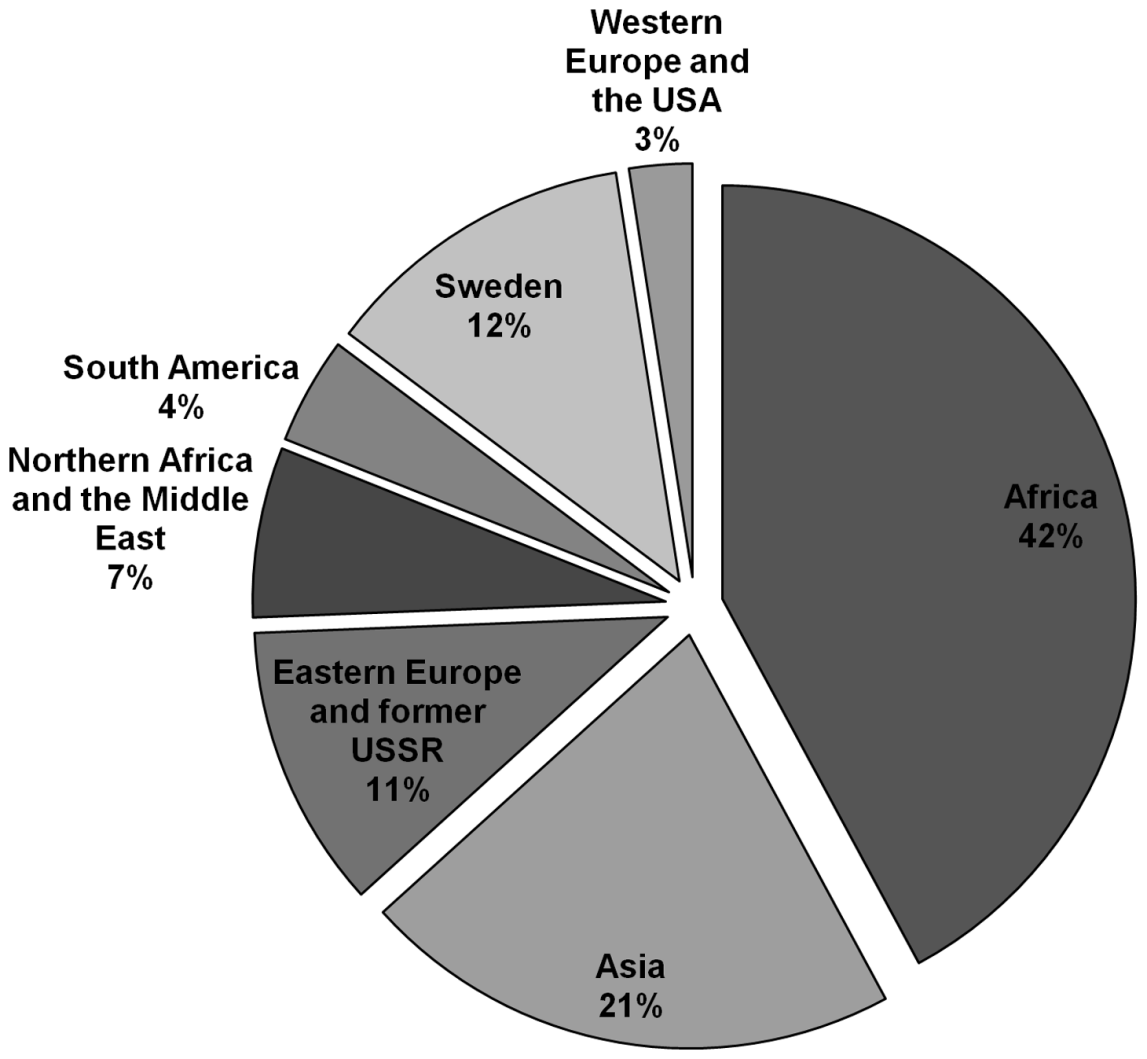
Tuberculosis cases confirmed by culture, Stockholm 2009–2011 by region of origin (n = 406).

In total, 406 isolates from 224 (55.2%) men and 182 (44.8%) women residing in the county of Stockholm during the years 2009–2011 were analyzed. Patients were between 2 and 93 years old with a median age of 39 years. Only 12% were of Swedish origin and the largest group were migrants from Africa (42%) and Asia (21%) (Figure 1). Of the 88% (n = 356) born outside of Sweden, the year of arrival was reported for 93% (n = 331) and 63% (n = 210) had lived in Sweden less than five years when being diagnosed with TB.

### Drug Resistance

Of the 406 isolates 64 were resistant to one or more of the drugs INH (n = 63), RIF (n = 13), EMB (n = 6) and PZA (n = 11). Eighteen isolates were resistant to more than one drug, and of those, 13 isolates were resistant to both INH and RIF which classified them as multidrug resistant (3.2%).

### Phylogenetic Lineages

In most cases, the isolates of foreign-born patients reflected genotypes common in their country of origin. For example, many of the patients infected with *Mtb* complex isolates belonging to the Beijing genotype were from Asia (42%) or Eastern Europe including the former USSR (34%). More than 40% (171/406) of the patients came from Africa, mainly the Horn of Africa (n = 143), and isolates from African patients were represented among all major lineages. There were six AFRI lineage isolates in our study and of these patients three came from the Gambia, two from Somalia and one from Morocco. The Beijing lineage, in which MIRU-VNTR is known to have less discriminatory power [9], [10] constituted 13% in our material. Table S1.

### Cluster Analysis

Spoligotyping of the isolates displayed 167 different patterns, of which 44 patterns were different clusters (cluster size 2–51 isolates). The largest cluster comprised 51 isolates and was of a Beijing genotype.

**Figure 2:**
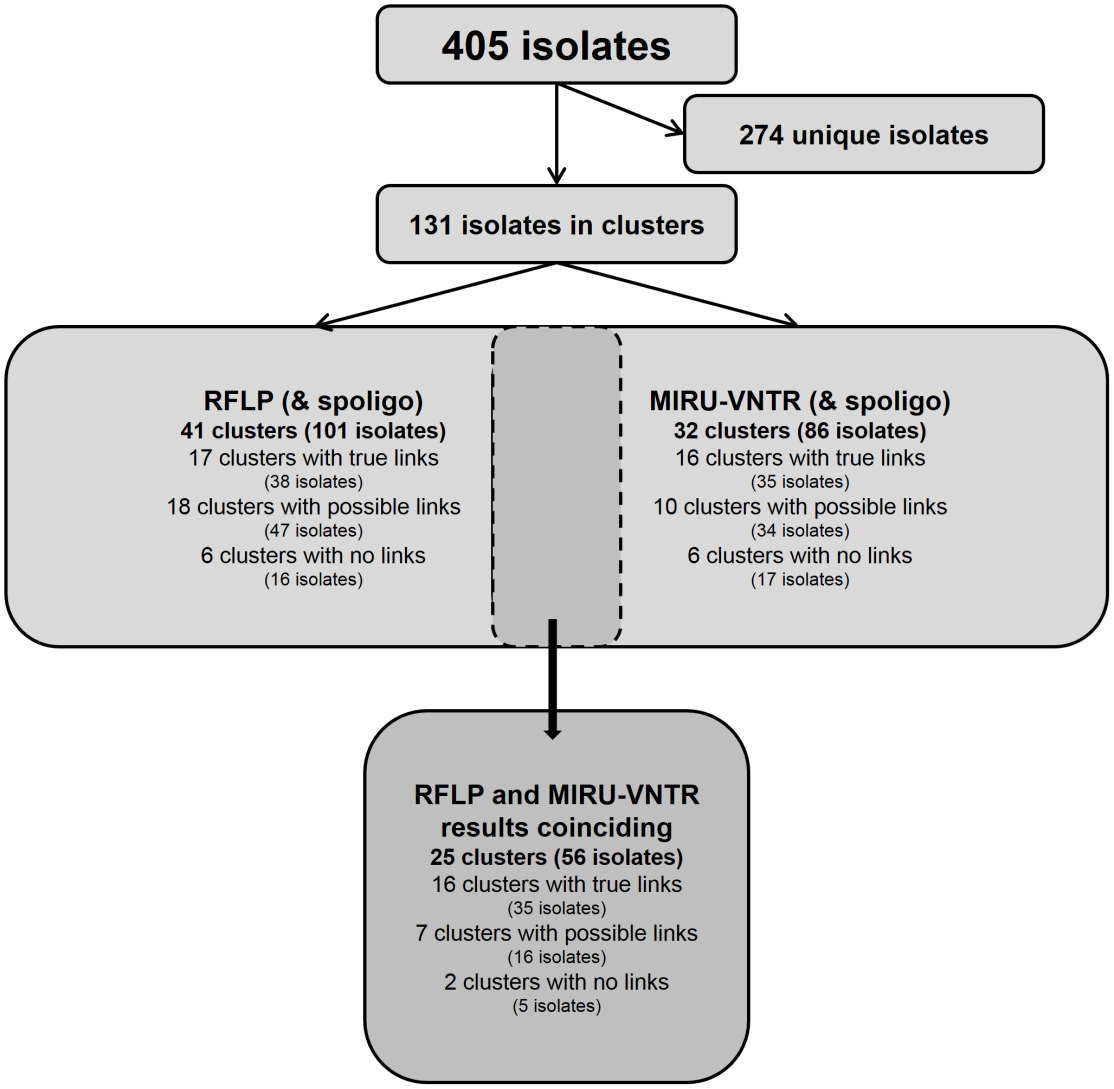
Number of *Mycobacterium tuberculosis* isolates clustering by different algorithms.

RFLP was not possible to perform on one isolate so RFLP results were available for 405 of the 406 isolates. For ten isolates that clustered in RFLP, the spoligo patterns did not match and they were consequently not considered to form a cluster. These isolates all had low IS*6110* copy numbers (five bands or less), reducing the discriminatory power of RFLP. In total, 15% of the 405 *Mtb* isolates (n = 60) had low IS*6110* copy numbers, and 31(50%) of these isolates were grouped in clusters, clearly demonstrating the reduced discriminatory power of RFLP. With RFLP, 304 isolates (75%) displayed unique patterns and 101 isolates (25%) belonged to 41 clusters containing 2–8 isolates each (average 2.24) (Figure 2). Of the 41 clusters 17 (38 isolates) were considered to have true links according to the epidemiological analysis. Of the remaining 24 clusters, 18 had possible links and six had no known links at all. In seven of these 24 clusters, the strains had low IS*6110* copy numbers.

MIRU-VNTR was performed on 406 isolates, out of which five displayed identical MIRU-VNTR patterns but not identical spoligo patterns and hence were not considered to form a cluster. Analyses comparing RFLP-results with MIRU-VNTR results were restricted to isolates with available data from both methods (n = 405). With MIRU-VNTR, 319 isolates (79%) were unique and 86 (21%) formed 32 different clusters containing 2–10 isolates (average 2.69). Out of these 32 clusters, 16 (35 isolates) had true links according to epidemiological analysis and of the other 16 clusters, 10 had possible links and 6 had no known links. In four of the clusters with no links the lineage was Beijing.

Combining RFLP (and spoligo) and MIRU-VNTR (and spoligo) gave 274 unique isolates (68%) while the remaining 131 isolates (32%) belonged to clusters by either one or both methods. For 56 (14%) isolates the clustering coincided with both methods and they formed 25 clusters comprising 2–4 isolates (average 2.24). In 16 of these 25 clusters, the cases had true links by conventional epidemiology. Seven of the remaining nine clusters with agreeing results in both RFLP and MIRU-VNTR had possible links due to shared geographical origin (Table 1) while two had no known links.

**Table 1:** Clustering by different algorithms and epidemiological links.

Typing method	Spoligo and IS*6110* RFLP andMIRU-VNTR combined	Spoligo and IS*6110* RFLP	Spoligo and MIRU-VNTR
**Number of cases clustered**	**56**	**101**	**86**
Clusters with true link	16 (64%)	17 (41%)	16 (50%)
Clusters with possible link	7 (28%)	19 (46%)	11 (34%)
Clusters with no link	2 (8%)	5 (12%)	5 (16%)
**Total number of clusters**	**25**	**41**	**32**

Apart from the 16 clusters with true links and coinciding RFLP and MIRU-VNTR results, there was one additional RFLP cluster with true epidemiological links where the MIRU-VNTR did not coincide. When comparing the MIRU-VNTR results in this cluster they differed in only one locus. In contrast, among the 18 RFLP clusters with only possible links and differing MIRU-VNTR results, a difference was seen in at least three loci or more.

The discriminatory power (DP) of each method and their combinations are visualized in Table 2 where they are showed by order of the Hunter-Gaston Index (HGI) of discriminatory ability. An index closer to one indicates higher DP.

**Table 2:** Typing methods and combinations in order by Hunter-Gaston index of discriminatory ability (n = 405) and cluster results.

Methods	HGI	No of uniquetypes (%)	No ofclusters	No of clusteredisolates (%)	Max no ofisolates	Average noof isolates
**spoligo**	0,9701	123 (30,4%)	44	282 (69,9%)	51	6,41
**RFLP**	0,9975	291 (71,9%)	41	114 (28,1%)	15	2,78
**MIRU-VNTR 24**	0,9985	312 (77%)	34	93 (23%)	10	2,74
**spoligo+MIRU-VNTR 24**	0,9986	319 (78,8%)	32	86 (21,2%)	10	2,69
**spoligo+RFLP**	0,9988	304 (75,1%)	41	101 (24,9%)	8	2,46
**spoligo+MIRU-VNTR 24+RFLP**	0,9995	349 (86,2%)	25	56 (13,8%)	4	2,24

Drug resistant isolates were not more prone to belong to a cluster in this study population. Out of 342 drug susceptible isolates, 107 (31%) belonged to a cluster in any or both of the methods compared to 64 isolates with some resistance out of which 21(33%) belonged to a cluster.

## Discussion

Our study showed that the DP of each method may vary depending on the strain and, not surprisingly, that a combination of both methods gave a more accurate result in better accordance with what could be confirmed by conventional contact tracing. However, the difference was not large enough to recommend the use of all methods in parallel. Since MIRU-VNTR is more convenient due to faster results and is less costly than RFLP, it should be the preferred method for routine genotyping with the option of complimentary genotyping when indicated.

As is logical spoligotyping alone had the lowest DP and a combination of all three methods had the highest (Table 2). In our material MIRU-VNTR alone had a higher DP than RFLP alone but when each method was combined with spoligo, RFLP performed slightly better. Even though less isolates were clustered by MIRU-VNTR, the clusters were larger.

The global concordance in isolates defined as unique or belonging to clusters amounted to 82% (n = 333) and of the remaining 72 isolates, 10% (n = 42) were clustered by RFLP only, seven percent (n = 27) belonging to clusters by MIRU-VNTR only and one percent (n = 3) belonging to a different cluster depending on method. This is very similar to the result in a recent study from the Netherlands which included ten times as many isolates [11].

A limitation of our study is that RFLP was the standard method for genotyping during the study period and the results were used in the contact investigations. This might have biased the number of true links confirmed by RFLP as compared to clusters with MIRU-VNTR which may not have been as thoroughly investigated for epidemiological links. Nevertheless one of the major drawbacks with RFLP is the time delay to obtain the results, and they were mainly used for retrospective confirmation of already suspected epidemiological links. A few unsuspected links were detected where cases clustered in time and space, i.e. turned out to be neighbors. In the clusters with possible links, the majority had the same geographical origin as the only connecting factor. As most were fairly recent immigrants this was interpreted as them being infected in their country of origin with highly genetically related strains.

The strength of our study was the completeness of epidemiological data available.

The number of isolates belonging to clusters when analyzed with IS*6110* RFLP (n = 101 (25%)) was higher than with MIRU-VNTR (n = 86 (21%)), however this may be explained by 31 of the 101 isolates belonging to clusters with RFLP having low IS*6110* copy numbers. Clusters identified with IS*6110* RFLP coincided slightly better with epidemiological links. The lower mean number of cases per cluster with RFLP also supports our theory that RFLP has a somewhat better DP than MIRU-VNTR except for strains with low IS*6110* copy numbers.

A recently published Dutch study [12] found the degree of clonality when typing with MIRU-VNTR, too high to apply a less strict definition of clustering. In our study there was one RFLP cluster with a true epidemiological link where the MIRU-VNTR results showed a single-locus variation. Even if the definition of clustering should be kept strict, single-locus variations do sometimes occur in isolates with definite links [13]. If a suspected link is not confirmed by MIRU-VNTR, it seems reasonable to still consider this possibility if the difference is only in a single locus.

When analyzing the MIRU-VNTR results, a total of 16 clusters had no known epidemiological link but 11 of these 16 clusters had a possible link through geographic origin in common. In four clusters with no connection, the isolates were of the Beijing genotype and belonged to clusters by MIRU-VNTR but not by RFLP. A true difference was further supported by the strains having different drug-susceptibility patterns in two of the clusters. It has previously been reported that the DP of MIRU-VNTR is reduced when it comes to isolates of the Beijing genotype [9], [14], and that this family of strains may require second-line typing by IS*6110* RFLP or hypervariable MIRU-VNTR loci [10], [13]. In contrast, the DP of MIRU-VNTR was better for isolates with few IS*6110* bands by RFLP. A German study published in 2011 [10] showed very similar results with false clustering by MIRU-VNTR of Beijing strains and by RFLP of strains with few IS*6110* RFLP bands. Due to the low DP of RFLP in isolates with few IS*6110* bands, a Danish study [15] of clustering in a low-burden country by analysis of nationwide genotyping with IS*6110* RFLP, excluded strains with less than five IS*6110* bands.

If RFLP has a higher DP, analysis with this method should result in a higher number of different patterns compared to MIRU-VNTR. However, in our material we had 15% low IS*6110* copy number isolates where RFLP is less precise. On the other hand, we also had 13% of the isolates belonging to the Beijing lineage in which MIRU-VNTR is known to be less discriminatory. Of the isolates that clustered only with RFLP, 52% were low IS*6110* copy number isolates and of the isolates that clustered only with MIRU-VNTR 33% were of the Beijing lineage, clearly illustrating the weakness of each method.

There was no indication of drug resistance influencing the likelihood of clustering in our material. In a Swedish context this is reasonable. Few cases would have inadequate treatment for very long since good laboratory facilities are country-wide and readily available and line probe assay provides information on possible resistance within a week. The largest cluster ever detected in Sweden was resistant to isoniazid [16], but the domestic spread was most likely more related to insufficient and complicated contact tracing [17] than to the strain being resistant.

## Conclusions

Genotyping is a useful tool to improve contact tracing and detect unknown connections, something that can be very helpful if the results are made available in a timely manner. A combination of all three methods resulted in confirmation of 16 of the 17 clusters with true links in Sweden.

In order to get a quick and reliable genotyping result a combination of spoligotyping and 24 loci MIRU-VNTR is sufficient in the majority of cases. Thus only Beijing lineage isolates might require an additional genotyping method, for better discriminatory power. We propose that if a link is suspected but not confirmed by MIRU-VNTR, the degree of difference should be investigated before a connection is discarded as not likely. Current genotyping techniques will only give guidance to investigate connections between clinical TB cases. If isolates from TB cases do not cluster, a direct true link can usually be ruled out unless the difference is minimal. On the other hand, clustering should be explored for possible connection but is no absolute evidence for cases having a true link. When whole genome sequencing is readily available, identification of clusters is likely to be more accurate.

## Supporting Information

Table S1 MIRU-VNTR based dendrogram with spoligotypes and lineages.
(DOCX)
