# Supplementary material for: Comparison between RFLP and MIRU-VNTR Genotyping of Mycobacterium tuberculosis Strains Isolated in Stockholm 2009 to 2011
Source: PLoS One. 2014 Apr 14;9(4):e95159. doi: 10.1371/journal.pone.0095159 (PMC3986374; doi:10.1371/journal.pone.0095159)
Supplement: Table S1 — MIRU-VNTR based dendrogram with spoligotypes and lineages. (DOCX) [file pone.0095159.s001.docx]

Haarlem

X

H37Rv

LAM

Ugandal

NEW-1

S

URAL

TUR

Beijing

Delhi/CAS

West African

EAI

Delhi/CAS

Linages

spoligotypes

MIRU-VNTR based dendrogram

Haarlem

X

H37Rv

LAM

Ugandal

NEW-1

S

URAL

TUR

Beijing

Delhi/CAS

West African

EAI

Delhi/CAS

H

T4, T1

T1

T1,T3, LAM

X1, T1, NIT

H4

T2, S

H4

T1, T3

Beijing

CAS

AFRI

EAI, NIT

CAS
